# Supplementary material for: Mental health among sexual and gender minorities: A Finnish population-based study of anxiety and depression discrepancies between individuals of diverse sexual orientations and gender minorities and the majority population
Source: PLoS One. 2022 Nov 4;17(11):e0276550. doi: 10.1371/journal.pone.0276550 (PMC9635740; doi:10.1371/journal.pone.0276550)
Supplement: S1 File — Detailed information on the categorization of textbox responses on the sexual orientation and gender identity questions. (DOCX) [file pone.0276550.s001.docx]

Supplement:

**Mental health among sexual and gender minorities: A Finnish population-based study of anxiety and depression discrepancies between individuals of diverse sexual orientations and gender minorities and the majority population**

Marianne Källström, Nicole Nousiainen, Patrick Jern, Sabina Nickull, and Annika Gunst

Department of Psychology, Åbo Akademi University, Finland

**Categorization of Textbox Responses on the Sexual Orientation and Gender Identity Questions**

The survey was conducted in Finnish, so all textbox responses were given in Finnish. All examples and citations of answers below have been made by the authors, so that the second author (N.N.) made translations which were then back translated and checked by the first and last authors (M.K. & A.G.). The terminology for sexual orientations in Finnish consists largely of straightforward and literal translations from English terms (e.g., the term “sukupuoleton” literally means “without gender” and corresponds to the term agender in English). In some cases English terms have even been adopted straight into the Finnish language (e.g., the term queer is used in Finnish and it is used in the same way as in English). No country-specific or culture-specific terms without English equivalents we mentioned in the answers provided by our participants.

**Sexual Orientation Responses**

Responses with multiple orientations (e.g., “asexual demisexual”) were categorized according to the first sexual orientation mentioned (*n* = 12). However, participants who described themselves as heterosexual as well as some other minority category (*n* = 3; e.g., “heterosexual asexual”), were coded according to the sexual minority stated, as we reasoned that these participants had deliberately not chosen the alternative “heterosexual” and had wanted to further define their orientation. Answers such as “mostly heterosexual”, “heterosexual but sometimes interested in people of the same sex”, and “bicurious” (*n* = 20), were, however, coded as heterosexual as these answers implied a heterosexual orientation. Some participants had also described their sexual as well as romantic preference (*n* = 8; e.g., “asexual demiromantic”). These were coded according to the sexual orientation mentioned. Answers such as “I am attracted to the person, regardless of their gender” were interpreted as pansexual (*n* = 3). Answers such as “I do not have a sexual orientation”, “I do not want to define my sexual orientation”, and “I do not know/ I am not sure” were included a category called *questioning/undefined* (*n* = 28).

**Gender Identity Responses**

We used the term binary transgender (*n* = 26) to collectively describe transgender women and transgender men. We applied the term non-binary to participants who described themselves as “non-binary” (*n* = 20), “bigender” (*n* = 1), “genderqueer” (*n* = 3), or “genderfluid” (*n* = 2). Participants who described themselves as “genderqueer woman” (*n* = 1), “non-binary femme” (*n* = 1), and “non-binary masculine” (*n* = 3) were also coded as non-binary, as were participants with answers such as “physically female but spiritually something in between” and “a bit of both” (*n* = 2). Finally, “demi” and “demi man” (*n* = 2) were also included in this category, as *demigender* is sometimes considered to describe non-binary gender identities (Adams & Vincent, 2019). We used the term agender for those who described their gender identity as “agender/no gender” (*n* = 12), “gender neutral” (*n =* 2), and “undefined” (*n* = 3).

**References**

Adams, N. J., & Vincent, B. (2019). Suicidal thoughts and behaviors among transgender adults in relation to education, ethnicity, and income: A systematic review. *Transgender Health*, *4*(1), 226–246. https://doi.org/10.1089/trgh.2019.0009
