# Supplementary material for: Mental health among sexual and gender minorities: A Finnish population-based study of anxiety and depression discrepancies between individuals of diverse sexual orientations and gender minorities and the majority population
Source: PLoS One. 2022 Nov 4;17(11):e0276550. doi: 10.1371/journal.pone.0276550 (PMC9635740; doi:10.1371/journal.pone.0276550)
Supplement: S2 File — (DOCX) [file pone.0276550.s002.docx]

Mental health among sexual and gender minorities: A Finnish population-based study of anxiety and depression discrepancies between individuals of diverse sexual orientations and gender minorities and the majority population

**Supplement 2: SPSS Syntax for the Statistical Analyses**

Marianne Källström, Nicole Nousiainen, Patrick Jern, Sabina Nickull, and Annika Gunst

Department of Psychology, Åbo Akademi University, Finland

**Differences in depression between heterosexual and sexual minority participants**

* Generalized Estimating Equations.

GENLIN Depressiontotal BY Sexual_orientation_new2 (ORDER=DESCENDING) WITH Age

/MODEL Sexual_orientation_new2 Age INTERCEPT=YES

DISTRIBUTION=NORMAL LINK=IDENTITY

/CRITERIA SCALE=MLE PCONVERGE=1E-006(ABSOLUTE) SINGULAR=1E-012 ANALYSISTYPE=3(WALD) CILEVEL=95

LIKELIHOOD=FULL

/EMMEANS TABLES=Sexual_orientation_new2 SCALE=ORIGINAL COMPARE=Sexual_orientation_new2

CONTRAST=PAIRWISE PADJUST=LSD

/REPEATED SUBJECT=FamilyID SORT=YES CORRTYPE=INDEPENDENT ADJUSTCORR=YES COVB=ROBUST

/MISSING CLASSMISSING=EXCLUDE

/PRINT CPS DESCRIPTIVES MODELINFO FIT SUMMARY SOLUTION GEF.

**Differences in anxiety between heterosexual and sexual minority participants**

* Generalized Estimating Equations.

GENLIN Anxietytotal BY Sexual_orientation_new2 (ORDER=DESCENDING) WITH Age

/MODEL Sexual_orientation_new2 Age INTERCEPT=YES

DISTRIBUTION=NORMAL LINK=IDENTITY

/CRITERIA SCALE=MLE PCONVERGE=1E-006(ABSOLUTE) SINGULAR=1E-012 ANALYSISTYPE=3(WALD) CILEVEL=95

LIKELIHOOD=FULL

/EMMEANS TABLES=Sexual_orientation_new2 SCALE=ORIGINAL COMPARE=Sexual_orientation_new2

CONTRAST=PAIRWISE PADJUST=LSD

/REPEATED SUBJECT=FamilyID SORT=YES CORRTYPE=INDEPENDENT ADJUSTCORR=YES COVB=ROBUST

/MISSING CLASSMISSING=EXCLUDE

/PRINT CPS DESCRIPTIVES MODELINFO FIT SUMMARY SOLUTION GEF.

**Differences in depression between cisgendered and gender minority participants**

* Generalized Estimating Equations.

GENLIN Depressiontotal BY Gender_identity_new (ORDER=DESCENDING) WITH Age

/MODEL Age Gender_identity_new INTERCEPT=YES

DISTRIBUTION=NORMAL LINK=IDENTITY

/CRITERIA SCALE=MLE PCONVERGE=1E-006(ABSOLUTE) SINGULAR=1E-012 ANALYSISTYPE=3(WALD) CILEVEL=95

LIKELIHOOD=FULL

/EMMEANS TABLES=Gender_identity_new SCALE=ORIGINAL COMPARE=Gender_identity_new CONTRAST=PAIRWISE

PADJUST=LSD

/REPEATED SUBJECT=FamilyID SORT=YES CORRTYPE=INDEPENDENT ADJUSTCORR=YES COVB=ROBUST

/MISSING CLASSMISSING=EXCLUDE

/PRINT CPS DESCRIPTIVES MODELINFO FIT SUMMARY SOLUTION GEF.

**Differences in anxiety between cisgendered and gender minority participants**

* Generalized Estimating Equations.

GENLIN Anxietytotal BY Gender_identity_new (ORDER=DESCENDING) WITH Age

/MODEL Age Gender_identity_new INTERCEPT=YES

DISTRIBUTION=NORMAL LINK=IDENTITY

/CRITERIA SCALE=MLE PCONVERGE=1E-006(ABSOLUTE) SINGULAR=1E-012 ANALYSISTYPE=3(WALD) CILEVEL=95

LIKELIHOOD=FULL

/EMMEANS TABLES=Gender_identity_new SCALE=ORIGINAL COMPARE=Gender_identity_new CONTRAST=PAIRWISE

PADJUST=LSD

/REPEATED SUBJECT=FamilyID SORT=YES CORRTYPE=INDEPENDENT ADJUSTCORR=YES COVB=ROBUST

/MISSING CLASSMISSING=EXCLUDE

/PRINT CPS DESCRIPTIVES MODELINFO FIT SUMMARY SOLUTION GEF.

**Differences in depression between the different sexual orientation groups**

* Generalized Estimating Equations.

GENLIN Depressiontotal BY Sexual_orientation_new (ORDER=DESCENDING) WITH Age

/MODEL Sexual_orientation_new Age INTERCEPT=YES

DISTRIBUTION=NORMAL LINK=IDENTITY

/CRITERIA SCALE=MLE PCONVERGE=1E-006(ABSOLUTE) SINGULAR=1E-012 ANALYSISTYPE=3(WALD) CILEVEL=95

LIKELIHOOD=FULL

/EMMEANS TABLES=Sexual_orientation_new SCALE=ORIGINAL COMPARE=Sexual_orientation_new

CONTRAST=PAIRWISE PADJUST=LSD

/REPEATED SUBJECT=FamilyID SORT=YES CORRTYPE=INDEPENDENT ADJUSTCORR=YES COVB=ROBUST

/MISSING CLASSMISSING=EXCLUDE

/PRINT CPS DESCRIPTIVES MODELINFO FIT SUMMARY SOLUTION.

**Differences in anxiety between the sexual orientation groups**

* Generalized Estimating Equations.

GENLIN Anxietytotal BY Sexual_orientation_new (ORDER=DESCENDING) WITH Age

/MODEL Sexual_orientation_new Age INTERCEPT=YES

DISTRIBUTION=NORMAL LINK=IDENTITY

/CRITERIA SCALE=MLE PCONVERGE=1E-006(ABSOLUTE) SINGULAR=1E-012 ANALYSISTYPE=3(WALD) CILEVEL=95

LIKELIHOOD=FULL

/EMMEANS TABLES=Sexual_orientation_new SCALE=ORIGINAL COMPARE=Sexual_orientation_new

CONTRAST=PAIRWISE PADJUST=LSD

/REPEATED SUBJECT=FamilyID SORT=YES CORRTYPE=INDEPENDENT ADJUSTCORR=YES COVB=ROBUST

/MISSING CLASSMISSING=EXCLUDE

/PRINT CPS DESCRIPTIVES MODELINFO FIT SUMMARY SOLUTION.

**Differences in depression between the gender identity groups**

* Generalized Estimating Equations.

GENLIN Depressiontotal BY Gender_identity_new3 (ORDER=DESCENDING) WITH Age

/MODEL Age Gender_identity_new3 INTERCEPT=YES

DISTRIBUTION=NORMAL LINK=IDENTITY

/CRITERIA SCALE=MLE PCONVERGE=1E-006(ABSOLUTE) SINGULAR=1E-012 ANALYSISTYPE=3(WALD) CILEVEL=95

LIKELIHOOD=FULL

/EMMEANS TABLES=Gender_identity_new3 SCALE=ORIGINAL COMPARE=Gender_identity_new3

CONTRAST=PAIRWISE PADJUST=LSD

/REPEATED SUBJECT=FamilyID SORT=YES CORRTYPE=INDEPENDENT ADJUSTCORR=YES COVB=ROBUST

/MISSING CLASSMISSING=EXCLUDE

/PRINT CPS DESCRIPTIVES MODELINFO FIT SUMMARY SOLUTION.

**Differences in anxiety between the gender identity groups**

* Generalized Estimating Equations.

GENLIN Anxietytotal BY Gender_identity_new3 (ORDER=DESCENDING) WITH Age

/MODEL Age Gender_identity_new3 INTERCEPT=YES

DISTRIBUTION=NORMAL LINK=IDENTITY

/CRITERIA SCALE=MLE PCONVERGE=1E-006(ABSOLUTE) SINGULAR=1E-012 ANALYSISTYPE=3(WALD) CILEVEL=95

LIKELIHOOD=FULL

/EMMEANS TABLES=Gender_identity_new3 SCALE=ORIGINAL COMPARE=Gender_identity_new3

CONTRAST=PAIRWISE PADJUST=LSD

/REPEATED SUBJECT=FamilyID SORT=YES CORRTYPE=INDEPENDENT ADJUSTCORR=YES COVB=ROBUST

/MISSING CLASSMISSING=EXCLUDE

/PRINT CPS DESCRIPTIVES MODELINFO FIT SUMMARY SOLUTION.

**Differences in depression between majority, single minority and double minority participants**

* Generalized Estimating Equations.

GENLIN Depressiontotal BY Minority_status (ORDER=DESCENDING) WITH Age

/MODEL Minority_status Age INTERCEPT=YES

DISTRIBUTION=NORMAL LINK=IDENTITY

/CRITERIA SCALE=MLE PCONVERGE=1E-006(ABSOLUTE) SINGULAR=1E-012 ANALYSISTYPE=3(WALD) CILEVEL=95

LIKELIHOOD=FULL

/EMMEANS TABLES=Minority_status SCALE=ORIGINAL COMPARE=Minority_status CONTRAST=PAIRWISE

PADJUST=LSD

/REPEATED SUBJECT=FamilyID SORT=YES CORRTYPE=INDEPENDENT ADJUSTCORR=YES COVB=ROBUST

/MISSING CLASSMISSING=EXCLUDE

/PRINT CPS DESCRIPTIVES MODELINFO FIT SUMMARY SOLUTION.

**Differences in anxiety between majority, single minority and double minority participants**

* Generalized Estimating Equations.

GENLIN Anxietytotal BY Minority_status (ORDER=DESCENDING) WITH Age

/MODEL Minority_status Age INTERCEPT=YES

DISTRIBUTION=NORMAL LINK=IDENTITY

/CRITERIA SCALE=MLE PCONVERGE=1E-006(ABSOLUTE) SINGULAR=1E-012 ANALYSISTYPE=3(WALD) CILEVEL=95

LIKELIHOOD=FULL

/EMMEANS TABLES=Minority_status SCALE=ORIGINAL COMPARE=Minority_status CONTRAST=PAIRWISE

PADJUST=LSD

/REPEATED SUBJECT=FamilyID SORT=YES CORRTYPE=INDEPENDENT ADJUSTCORR=YES COVB=ROBUST

/MISSING CLASSMISSING=EXCLUDE

/PRINT CPS DESCRIPTIVES MODELINFO FIT SUMMARY SOLUTION.
