# Supplementary material for: Mental health among sexual and gender minorities: A Finnish population-based study of anxiety and depression discrepancies between individuals of diverse sexual orientations and gender minorities and the majority population
Source: PLoS One. 2022 Nov 4;17(11):e0276550. doi: 10.1371/journal.pone.0276550 (PMC9635740; doi:10.1371/journal.pone.0276550)
Supplement: S1 Table — (DOCX) [file pone.0276550.s003.docx]

**Mental health among sexual and gender minorities: A Finnish population-based study of anxiety and depression discrepancies between individuals of diverse sexual orientations and gender minorities and the majority population**

Marianne Källström, Nicole Nousiainen, Patrick Jern, Sabina Nickull, and Annika Gunst

Department of Psychology, Åbo Akademi University, Finland

Supporting material 3: Tables

| **Table A**  *Pairwise Comparisons of Depression Symptom Scores by Gender Identity* | | | | | |
| --- | --- | --- | --- | --- | --- |
| Comparison group  (A) | Other identities  (B) | Mean difference  (A - B) | *SE* | *p* | 95% CI |
| Binary Transgender | Cisgender | 1.53 | 1.09 | .161 | [-0.61, 3.66] |
|  | Nonbinary | -2.77 | 1.43 | .053 | [-5.57, 0.04] |
|  | Agender | 0.09 | 1.81 | .958 | [-3.45, 3.64] |
|  |  |  |  |  |  |
| Nonbinary | Cisgender | 4.29 | 0.91 | < .001*** | [2.51, 6.08] |
|  | Binary Transgender | 2.77 | 1.43 | .053 | [-0.04, 5.57] |
|  | Agender | 2.86 | 1.77 | .105 | [-0.60, 6.32] |
|  |  |  |  |  |  |
| Agender | Cisgender | 1.43 | 1.49 | .338 | [-1.49, 4.36] |
|  | Binary Transgender | -0.09 | 1.81 | .958 | [-3.64, 3.45] |
|  | Nonbinary | -2.86 | 1.77 | .105 | [-6.32, 0.60] |
| *Note.* Pairwise comparisons of mean differences in depression rates from the Generalized Estimating Equations analysis. *SE=* standard error. CI = confidence interval. * *p* < .05. ** *p* < .01. *** *p* < .001 | | | | | |

| **Table B** *Pairwise Comparisons of Anxiety Symptom Scores by Gender Identity* | | | | | |
| --- | --- | --- | --- | --- | --- |
| Comparison group  (A) | Other orientations (B) | Mean difference  (A - B) | *SE* | *p* | 95% CI |
| Binary Transgender | Cisgender | 0.99 | 0.87 | .259 | [-0.73, 2.70] |
|  | Nonbinary | -3.67 | 1.34 | .006** | [-6.29,-1.05] |
|  | Agender | -0.30 | 1.65 | .856 | [-3.54, 2.94] |
| Nonbinary | Cisgender | 4.66 | 1.03 | < .001*** | [2.65, 6.67] |
|  | Binary Transgender | 3.67 | 1.34 | .006** | [1.05, 6.29] |
|  | Agender | 3.37 | 1.69 | .046* | [0.05, 6.69] |
| Agender | Cisgender | 1.29 | 1.39 | .353 | [-1.43, 4.01] |
|  | Binary Transgender | 0.30 | 1.65 | .856 | [-2.94, 3.54] |
|  | Nonbinary | -3.37 | 1.69 | .046* | [-6.69, -0.05] |
| *Note.* Pairwise comparisons of mean differences in anxiety rates from the Generalized Estimating Equations analysis. *SE=* standard error. CI = confidence interval. * *p* < .05. ** *p* < .01. *** *p* < .001. | | | | | |
