# Supplementary material for: Mental health among sexual and gender minorities: A Finnish population-based study of anxiety and depression discrepancies between individuals of diverse sexual orientations and gender minorities and the majority population
Source: PLoS One. 2022 Nov 4;17(11):e0276550. doi: 10.1371/journal.pone.0276550 (PMC9635740; doi:10.1371/journal.pone.0276550)
Supplement: Attachment — Submitted filename: Rebuttal letter.docx [file pone.0276550.s004.docx]

Rebuttal letter

March 31^st^, 2022

*PLOS ONE*

We thank you for the comments raised by our manuscript (PONE-D-22-02804). We would also like to apologize for not adding a rebuttal letter or a manuscript version with highlighted changes, as Hilary Genn Givero kindly pointed out in her e-mail on March 23rd 2022. We hope this rebuttal letter and the newly uploaded highlighted version of our manuscript will make it possible to evaluate our work properly.

Below are our responses to the points raised by the academic editor Dr. Jakob Pietschnig in his rejection letter from Febryary 17^th^ 2022. We hope that we have addressed them in a satisfying way and that the manuscript will be now suited for peer review.

*“* *Dear Dr. Källström,*

*Thank you for submitting your manuscript to PLOS ONE. After careful consideration, we have decided that your manuscript does not meet our criteria for publication and must therefore be rejected.*

*Specifically:*

*The reported sample sizes in your text and tables do not add up (see for instance Table 1). Due to the low expected cell counts according to your research question, this may have a huge impact on your outcomes. Please carefully recheck all your calculations and outcomes according to their accuracy and provide the syntax for the analysis in a supplementary file. If you do so, I would be happy to reconsider your paper for publication as a new submission.”*

We sincerely thank you for pointing out these errors, as they are indeed vitally important considering the small group sizes in some of the analyzed participant groups. Below is a list of the changes we made to the manuscript after having carefully checked our analyses, SPSS outputs and the manuscript itself.

1. P. 12, Table 1:
   Two numbers had switched places with each other in Table 1. More specifically, the true number or participants who identify as queer was 3 and the number of participants who identify as demisexual was 8, and not vice versa. Consequently, we did not have to repeat or correct any of our analyses or change any conclusions in the manuscript, as this error only existed in Table 1 and not in the data the analyses are based on.
2. P. 15, Line 287:
   We added a sentence referring to the new syntax supplement: “The syntax for the analyses can be found in the Online Supplement (S2). “
3. P. 16, Line 302:
   Mean score changed: 11.96 🡪 11.97
   While carefully rechecking our analyses, outputs, tables, and text, we noted several minor rounding errors and errors in significance notation that had occurred when we transferred the results from our SPSS outputs to our manuscript text and tables. We corrected the rounding of these numbers to correspond to the exact results in our SPSS output. None of these changes resulted in any alterations concerning the interpretation of results of the conclusions of the study, as the rounding errors were very small and the errors in *p*-notation did not dilute any of the observed effects. We have listed these corrections below in order to make it easier to understand the changes in the highlighted manuscript version.
4. P. 16, Line 306:
   Rounding error corrected
5. P. 17, Table 2:
   Rounding errors corrected for the means for depression (heterosexual, cisgender, and bisexual participants) and anxiety (heterosexual, sexual minority, and gay/lesbian participants).
6. P. 18, Table 3:
   Rounding errors corrected for three *p*-values, also resulting in minor changes in the significance notations (depression of sexual minorities collectively, anxiety for sexual minorities collectively, anxiety for gay/lesbian participants).
7. P.19, Lines 232 and 327:
   Rounding errors corrected (See Table 3 and comment 6 above)
8. P.20, Table 4:
   a. Rounding errors corrected for two *p*-values and the significance notations corrected for two *p*-values.
   b. One confidence interval had typing errors (gay/lesbian vs EI) that were corrected (minus notation was missing for some of the values, so some CIs were reported as overlapping zero when they were in fact negative).
9. P. 21, Table 5:
   a. Rounding errors corrected for two *p*-values, resulting in minor changes in the significance notations (bisexual vs heterosexual and bisexual vs gay/lesbian).
   b. The confidence interval had a rounding error for the comparison between gay/lesbian and bisexual and for the comparisons between gay/lesbian and emerging identity.
10. P. 22, Line 345:
    Rounding error corrected (depression mean for cisgender participants)
11. P. 23, Table 6:
    a. Table heading changed: The word “gender minorities” was changed to gender minority individuals” in accordance with APA guidelines for bias-free language. This change was not asked for by the editor, but we took the liberty to change this term as we found it is probably good to alter it in order to comply to the common language and form standards in psychological research.
    b. One rounding error in a chi-squared value was corrected (depression for the agender participant group)
12. P. 27, 380:
    One rounding error in a mean value was corrected (depression among the double minority status participants).
13. P. 41, Line 712:
    We added a mention of the new supplementary syntax file containing the SPSS code for our analyses. We were not planning to publish the data because of the sensitivity of our research topic, but we would be happy to share it with editors or potential reviewers if there is anything about our analyses or the data itself that requires further clarification.

Thank you for your feedback and for the generous invitation to resubmit our manuscript. We are delighted to resubmit our work to PLOS ONE*,* and we hope you find the manuscript suitable for publication. Should any matter require further attention from me, please do not hesitate to contact me.

Sincerely,

On behalf of all authors,

Marianne Källström

MA (Psych), Doctoral candidate

Department of Psychology, Åbo Akademi University, 20500 Åbo, Finland.

E-mail: [mkallstr@abo.fi](mailto:mkallstr@abo.fi)

Telephone: +358 404170237
