# Supplementary material for: Mental health among sexual and gender minorities: A Finnish population-based study of anxiety and depression discrepancies between individuals of diverse sexual orientations and gender minorities and the majority population
Source: PLoS One. 2022 Nov 4;17(11):e0276550. doi: 10.1371/journal.pone.0276550 (PMC9635740; doi:10.1371/journal.pone.0276550)
Supplement: Attachment — Submitted filename: Response to Reviewers.docx [file pone.0276550.s006.docx]

September 23rd, 2022

Response to review comments

Manuscript title: Mental health among sexual and gender minorities: A Finnish population-based study of anxiety and depression discrepancies between individuals of diverse sexual orientations and gender minorities and the majority population

Manuscript number: PONE-D-22-08353

*Dear editor and dear reviewers,*

*Thank you once again for your feedback on our manuscript. We are happy to hand in this revised version that we feel corresponds to the changes asked for in the previous round of reviews. Below you can find a list of the comments we have received and how we have addressed them. Should we have missed anything or should any part or the manuscript still require changes, we will gladly address them and make further improvements. In addition, if anything seems to need further clarification on our part, we will do our best to answer whatever questions the manuscript may raise.*

Journal Requirements:

Please review your reference list to ensure that it is complete and correct. If you have cited papers that have been retracted, please include the rationale for doing so in the manuscript text, or remove these references and replace them with relevant current references. Any changes to the reference list should be mentioned in the rebuttal letter that accompanies your revised manuscript. If you need to cite a retracted article, indicate the article’s retracted status in the References list and also include a citation and full reference for the retraction notice.

*Response: Thank you for bringing this up. We went through the references section with special attention, and we did find a number of small formatting and spelling errors which we have now corrected. The changes are listed below. We believe that the reference list is now complete and correct, but if there is anything in it that seems to still require revisions we will of course go through the list again and make the required changes.*

*Reference 1: We changed the format of the reference to match the guidelines for PlosONE.*

*Reference 16: We deleted an unnecessary period from the end of the doi-number.*

*Reference 20: We changed the format of the reference to match the guidelines for PlosONE.*

*Reference 24: We realized that the doi-number of the reference and the name of the first author were correct, but not the other details. This means that we intended to refer to a another, slightly differently themed article written by M. Paz Galupo (published in another journal). We corrected the reference information and left the first author and the doi-number as they were. We sincerely apologize for this mistake.*

*Reference 28: We changed the formatting of the reference to match the guidelines for PlosONE (according to the instructions on how to cite a monograph on the internet).*

*Reference 29: We added a missing page number and a period to the reference.*

*Reference 35: We corrected the capitalization of the word “national” (National –> national)*

*Reference 37: We corrected the abbreviation of the journal name.*

*Reference 43: We deleted an unnecessary period from the end of the doi-number and we corrected the formatting of the author names.*

*Reference 48: We added a missing page number.*

*Reference 50: We corrected the spelling of the last author’s name.*

*Reference 53: We corrected two errors in punctuation.*

*Reference 56: We corrected a spelling error and added a missing comma.*

*Reference 61: We corrected the formatting so that it corresponds to the instructions for referring to a monograph on the internet.*

*Reference 62: We corrected the formatting of the reference.*

*Reference 63: We corrected the formatting of the reference and corrected the authors and the doi-number.*

*Reference 67: We corrected the formatting regarding punctuation and capitalization of the study title.*

*Reference 69: We corrected the formatting of the reference so that it corresponds to the instructions on how to refer to a monograph on the internet.*

*References 70-72 were added because of the changes we made based on Reviewer 1’s comments (please see comment 3 below). This means that the manuscript’s reference section now features 74 references in total (the total number of references in the previous version was 71).*

Review Comments to the Author

Reviewer #1: Thank you for your work to address my previous comments. I believe the manuscript has been improved as a result of the authors' responses to mine and other reviewers' comments.

As a side note, it looks like something happened with formatting of the manuscript such that the line numbers the authors gave for revisions were inaccurate. In any future revisions, it would be helpful to ensure that the line numbers in the responses to reviewer comments are consistent with where changes are located in the manuscript.

*Response: Thank you, we are delighted to hear that the changes we have made based on the previous comments seem to have improved the quality of the manuscript. We would also like to apologize for the mix-up with the line numbers. We will pay special attention to this in the future.*

Here are the few additional comments that authors should address:
1. The paragraph on page 11 is very long. Please divide into multiple paragraphs.

*Response: We divided the original paragraph in three, and we also found one error in punctuation that we corrected (“A subset of the invited individuals (n = 7,716) had previously participated in similar data collections by the same research group and had consented to being invited to participate in future studies [56], Despite some”). The comma after the word “studies” was changed into a period (“.”). Please see page 11 in the manuscript.*

2. The paragraph on pages 16-17 is also very long. Please divide into multiple paragraphs.

Response: *We divided the original paragraph into three shorter paragraphs (please see p 16-17).*

3. The authors noted in a response to another comment in the original revision that the population of Finland is homogenous in regard to race/ethnicity, education, and income level. I think this information would be important to include in the discussion of how the results of this study should be interpreted, particularly given that the authors discuss the broader implications of intersectionality on page 32. The homogeneity of the Finnish population would seem to indicate that the results are most generalizable to a similar population.

*Response: We agree, it would have been good to add these points into the manuscript itself as well. We added these points of discussion to the beginning of the Strengths and Limitations section (please see p 33).*

Reviewer #2: I thank the authors for their attention to my concerns. No further changes are sought by me in respect of this manuscript.

*Response: Thank you for your feedback, we really appreciate your work with reviewing this manuscript and helping us improve it.*

*Thank you once again for your valuable comments and for the opportunity to have our work reviewed at Plos One.*

*Sincerely, on behalf of my co-authors,*

*Marianne Källström*
